# Supplementary material for: Immunogenicity, Safety, and Anti-Viral Efficacy of a Subunit SARS-CoV-2 Vaccine Candidate in Captive Black-Footed Ferrets (Mustela nigripes) and Their Susceptibility to Viral Challenge
Source: Viruses. 2022 Oct 4;14(10):2188. doi: 10.3390/v14102188 (PMC9612080; doi:10.3390/v14102188)
Supplement: Supplementary file 1 [file viruses-14-02188-s001.zip › viruses-1889102-supplementary.pdf]

Immunogenicity, safety, and anti-viral efficacy of a subunit SARS-CoV-2 vaccine candidate in captive black-footed ferrets (*Mustela nigripes*) and their susceptibility to viral challenge.

Ariel E. Leon [1], Della Garelle [2], Airn Hartwig [3], Elizabeth A. Falendysz [1], Hon S. Ip [1], Julia S. Lankton [1], Tyler N. Tretten [2], Terry R. Spraker [4], Richard Bowen [3,†], Tonie E. Rocke [1,*,†]

Supplementary Materials

Table S1. Weights of transgenic mice that received passive transfers of black-footed ferret (*Mustela nigripes;* BFF) serum either from vaccinated (positive serum) or unvaccinated against SARS-CoV-2 BFF (negative serum), a monoclonal anti-S1 protein (monoclonal AB) or a sham transfer (negative) and then subsequently challenged with SARS-CoV-2. A subset of animals had adverse reactions to the passive transfers of BFF serum, which were euthanized.

				**weight (g) by day post-inoculation**						
**Cage**	**Mouse ID**	**BFF ID**	**treatment**	**day -1**	**day 0**	**day 1**	**day 2**	**day 3**	**day 4**	**day 5**
1	1	8593 -3	positive serum	19.52	18.24	18.5	18.86	19.37	19.81	19.74
1	2	8593 -12	positive serum	20.79	20.21	20.53	21.52	21.34	21.95	21.78
1	3	8847 -2	positive serum	22.09	22.21	20.40	19.67	18.93	18.74	17.98
1	4	8847 -12	positive serum	20.20	22.59	23.01	23.41	23.40	24.51	24.89
2	5	8995 -2	positive serum	20.32	**euthanized due to adverse reaction from serum transfer**					
2	6	8995 -12	positive serum	20.80	20.25	20.08	20.42	21.44	22.25	20.65
2	7	9135 -2	positive serum	23.39	**euthanized due to adverse reaction from serum transfer**					
2	8	9135 -12	positive serum	21.05	20.76	20.98	21.62	22.39	22.62	22.36
3	9	9189 -3	positive serum	19.87	19.10	19.30	20.23	20.22	20.41	20.56
3	10	9189 -12	positive serum	18.00	17.09	17.99	18.05	17.84	17.62	17.00
3	11	9219 -3	positive serum	21.16	21.08	19.72	19.46	18.81	18.83	19.31
3	12	9219 -12	positive serum	20.05	19.30	18.25	17.87	17.07	17.51	18.05

Immunogenicity, safety, and anti-viral efficacy of a subunit SARS-CoV-2 vaccine candidate in captive black-footed ferrets (*Mustela nigripes*) and their susceptibility to viral challenge.

Ariel E. Leon [1], Della Garelle [2], Airn Hartwig [3], Elizabeth A. Falendysz [1], Hon S. Ip [1], Julia S. Lankton [1], Tyler N. Tretten [2], Terry R. Spraker [4], Richard Bowen [3,†], Tonie E. Rocke [1,*,†]

4	13	9337 -2	positive serum	17.98	**euthanized due to adverse reaction from serum transfer**					
4	14	9337 -12	positive serum	21.48	21.2	19.9	19.96	20.44	20.82	20.03
4	15	9371 -3	positive serum	20.49	**euthanized due to adverse reaction from serum transfer**					
4	16	9371 -12	positive serum	18.43	17.49	17.15	17.71	18.69	19.20	19.28
5	17	9422 -3	positive serum	22.08	21.12	20.65	21.02	21.45	22.06	22.01
5	18	9422 -12	positive serum	21.25	19.80	20.08	20.64	20.93	21.43	20.40
5	19	9469 -3	positive serum	19.49	17.48	17.55	17.88	18.5	18.56	18.25
5	20	9469 -12	positive serum	21.12	20.72	21.35	21.49	21.51	21.71	20.62
6	21	9476 -2	positive serum	22.45	20.42	21.07	21.2	21.98	22.35	22.3
6	22	9476 -12	positive serum	21.66	20.02	20.15	20.90	21.71	21.81	22.03
6	23	9568 -3	positive serum	21.05	20.24	20.45	20.90	21.45	21.18	21.66
6	24	9568 -12	positive serum	18.69	19.26	19.39	19.45	19.97	19.96	20.07
7	25	9571 -2	positive serum	22.71	20.20	21.18	21.9	22.43	23.18	23.42
7	26	9571 -12	positive serum	23.14	23.27	24.39	24.12	24.62	24.88	23.11
7	27	9576 -3	positive serum	21.03	**euthanized due to adverse reaction from serum transfer**					
7	28	9576 -12	positive serum	20.16	20.52	20.99	21.25	21.52	21.65	21.16
8	29	9598 -3	positive serum	21.28	20.38	20.53	21.18	21.96	22.08	22.17

Immunogenicity, safety, and anti-viral efficacy of a subunit SARS-CoV-2 vaccine candidate in captive black-footed ferrets (*Mustela nigripes*) and their susceptibility to viral challenge.

Ariel E. Leon [1], Della Garelle [2], Airn Hartwig [3], Elizabeth A. Falendysz [1], Hon S. Ip [1], Julia S. Lankton [1], Tyler N. Tretten [2], Terry R. Spraker [4], Richard Bowen [3,†], Tonie E. Rocke [1,*,†]

8	30	9598 -12	positive serum	22.47	20.27	22.65	22.10	22.34	22.67	20.78
9	31	8710 -3	negative serum	21.30	19.16	20.02	20.02	20.66	20.66	20.91
9	32	8710 -12	negative serum	22.37	21.15	21.50	22.16	22.18	23.65	22.00
10	33	8721 -3	negative serum	20.38	**euthanized due to adverse reaction from serum transfer**					
10	34	8721 -12	negative serum	22.85	22.07	22.35	22.78	23.52	24.57	23.12
10	35	8970 -2	negative serum	18.20	16.44	17.25	17.63	18.27	19.01	18.70
10	36	8970 -12	negative serum	23.33	23.30	23.9	24.04	25.54	25.67	25.91
11	37	9247 -3	negative serum	20.45	**euthanized due to adverse reaction from serum transfer**					
11	38	9247 -12	negative serum	22.87	23.44	23.4	23.42	23.70	23.48	24.43
11	39	9311 -2	negative serum	16.44	**euthanized due to adverse reaction from serum transfer**					
11	40	9311 -12	negative serum	21.78	22.70	21.83	22.60	23.62	23.77	23.20
12	41	9388 -3	negative serum	20.30	**euthanized due to adverse reaction from serum transfer**					
12	42	9388 -12	negative serum	18.09	**euthanized due to adverse reaction from serum transfer**					
12	43	9425 -3	negative serum	18.10	**euthanized due to adverse reaction from serum transfer**					
12	44	9425 -12	negative serum	19.61	**euthanized due to adverse reaction from serum transfer**					
13	45	9519 -3	negative serum	22.08	**euthanized due to adverse reaction from serum transfer**					
13	46	9519 -12	negative serum	18.85	19.08	18.46	19.28	19.25	20.03	19.61

Immunogenicity, safety, and anti-viral efficacy of a subunit SARS-CoV-2 vaccine candidate in captive black-footed ferrets (*Mustela nigripes*) and their susceptibility to viral challenge.

Ariel E. Leon [1], Della Garelle [2], Airn Hartwig [3], Elizabeth A. Falendysz [1], Hon S. Ip [1], Julia S. Lankton [1],
Tyler N. Tretten [2], Terry R. Spraker [4], Richard Bowen [3,†], Tonie E. Rocke [1,*,†]

13	47	9592 -3	negative serum	21.26	20.13	20.28	20.54	21.13	22.00	22.25
13	48	9592 -12	negative serum	21.38	20.75	21.82	22.22	22.2	22.72	22.98
14	49	20 ug Ab	monoclonal AB	21.45	21.33	21.30	21.42	22.2	21.58	21.96
14	50	20 ug Ab	monoclonal AB	19.56	20.36	20.95	21.49	21.82	21.23	21.60
14	51	20 ug Ab	monoclonal AB	21.49	21.93	22.18	21.98	22.27	22.01	22.13
14	52	20 ug Ab	monoclonal AB	18.81	19.74	20.02	20.39	20.55	21.22	20.43
15	53	PBS	negative	22.21	21.45	21.89	21.89	22.49	22.57	22.81
15	54	PBS	negative	23.55	23.20	23.80	23.33	23.63	24.24	24.23
15	55	PBS	negative	22.60	23.41	23.20	23.35	23.73	23.86	23.72
15	56	PBS	negative	23.30	23.46	24.31	23.98	24.63	25.16	24.36

Immunogenicity, safety, and anti-viral efficacy of a subunit SARS-CoV-2 vaccine candidate in captive black-footed ferrets (*Mustela nigripes*) and their susceptibility to viral challenge.

Ariel E. Leon [1], Della Garelle [2], Airn Hartwig [3], Elizabeth A. Falendysz [1], Hon S. Ip [1], Julia S. Lankton [1], Tyler N. Tretten [2], Terry R. Spraker [4], Richard Bowen [3,†], Tonie E. Rocke [1,*,†]

Table S2. Histology results in mice that received positive serum (PS) from black-footed ferrets (*Mustela nigripes*; BFF) vaccinated against SARS-CoV-2, negative serum (NS) from unvaccinated BFF, or monoclonal antibody (MAB) against SARS-CoV-2 and were then challenged with SARS-CoV-2. Negative control animals received phosphate buffered saline but were not challenged. BH=brain hemorrhage; PVC=perivascular cuffing; GL=gliosis, LH=lung hemorrhage, LPI= lung perivascular inflammation, IP= interstitial pneumonia, LE= lung edema.

ID	Treat-ment	BH	BH count	PVC	PVC count	GL	LH	LPI	IP	IP %	LE	Brain Grade	Lung Grade	Total Grade
1	PS	+	2	-		-	+	+	+	<10	-	0	1	1
2	PS	-		-		-	+	+	+	<10	-	0	1	1
3	PS	+	1	+	7	+	+	+	+	20-40%	-	1	3	4
4	PS	+	3	-		-	+	-	-		-	0	0	0
6	PS	-		+	16	+	+	-	-		-	2	0	2
8	PS	-		-		-	+	+	+	20-40%	-	0	3	3
9	PS	+	2	-		-	+	+	+	10-20%	-	0	2	2
10	PS	-		-		-	+	+	+	40-50%	+	0	4	4
11	PS	+	3	-		-	+	+	+	<10	-	0	1	1
12	PS	+	3	-		-	+	-	+	10-20%	-	0	2	2
14	PS	-		-		-	+	+	+	20-40%	-	0	3	3
16	PS	+	2	-		-	+	+	+	20-40%	+	0	3	3
17	PS	+	2	-		-	+	+	-		-	0	0	0
18	PS	+	2	+	14	+	+	+	+	<10	-	2	1	3
19	PS	-		+	2	+	+	+	-		-	1	0	1

Immunogenicity, safety, and anti-viral efficacy of a subunit SARS-CoV-2 vaccine candidate in captive black-footed ferrets (*Mustela nigripes*) and their susceptibility to viral challenge.

Ariel E. Leon [1], Della Garelle [2], Airn Hartwig [3], Elizabeth A. Falendysz [1], Hon S. Ip [1], Julia S. Lankton [1],
Tyler N. Tretten [2], Terry R. Spraker [4], Richard Bowen [3,†], Tonie E. Rocke [1,*,†]

20	PS	-		+	9	+	+	-	-		-	1	0	1
21	PS	-		-		-	+	+	+	<10	-	0	1	1
22	PS	+	1	-		-	+	+	+	>50	-	0	5	5
23	PS	-		-		-	+	+	+	40-50%	+	0	4	4
24	PS	-		-		-	+	+	+	20-40%	+	0	3	3
25	PS	+	1	-		-	+	+	+	<10	-	0	1	1
26	PS	-		-		-	+	+	+	>50	+	0	5	5
28	PS	+	1	-		-	+	+	+	>50	+	0	5	5
29	PS	-		+	28	-	-	+	+	<10	-	3	1	4
30	PS	+	2	-		-	+	+	+	<10	-	0	1	1
**Mean**			**1.9**		**12.7**							**0.4**	**2**	**2.4**
31	NS	+	2	-		-	+	+	+	>50	+	0	5	5
32	NS	+	3	+	2	+	+	+	+	<10	-	1	1	2
34	NS	-		+	4	+	+	-	-		-	1	0	1
35	NS	-		+	12	-	+	+	+	20-40%	+	2	3	5
36	NS	-		-		-	-	+	+	<10	-	0	1	1
38	NS	-		-		-	+	+	+	<10	-	0	1	1
40	NS	-		-		-	+	+	+	<10	-	0	1	1
46	NS	-		-		-	+	+	+	40-50%	+	0	4	4
47	NS	+	3	-		-	+	+	+	20-40%	+	0	3	3
48	NS	+	6	-		-	+	+	+	20-40%	+	0	3	3

Immunogenicity, safety, and anti-viral efficacy of a subunit SARS-CoV-2 vaccine candidate in captive black-footed ferrets (*Mustela nigripes*) and their susceptibility to viral challenge.

Ariel E. Leon [1], Della Garelle [2], Airn Hartwig [3], Elizabeth A. Falendysz [1], Hon S. Ip [1], Julia S. Lankton [1], Tyler N. Tretten [2], Terry R. Spraker [4], Richard Bowen [3,†], Tonie E. Rocke [1,*,†]

**Mean**			**3.5**		**6**							**0.4**	**2**	**3**
49	MAB	-		-		-	+	+	+	<10	+	0	1	1
50	MAB	-		-		-	+	-	+	<10	-	0	1	1
51	MAB	-		-		-	+	+	+	<10	-	0	1	1
52	MAB	+	5	-		-	+	+	+	<10	-	0	1	1
**Mean**			**5**									**0**	**1**	**1**
53	PBS	+	1	-		-	+	-	-		-	0	0	0
54	PBS	+	1	-		-	+	-	-		-	0	0	0
55	PBS	+	1	-		-	+	-	-		-	0	0	0
56	PBS	+	3	-		-	+	-	-		-	0	0	0
**Mean**			**1.5**									**0**	**0**	**0**

Immunogenicity, safety, and anti-viral efficacy of a subunit SARS-CoV-2 vaccine candidate in captive black-footed ferrets (*Mustela nigripes*) and their susceptibility to viral challenge.

Ariel E. Leon [1], Della Garelle [2], Airn Hartwig [3], Elizabeth A. Falendysz [1], Hon S. Ip [1], Julia S. Lankton [1], Tyler N. Tretten [2], Terry R. Spraker [4], Richard Bowen [3,†], Tonie E. Rocke [1,*,†]

Table S3. Histology grades in mice that received positive serum (PS) from black-footed ferrets (*Mustela nigripes*; BFF) vaccinated against SARS-CoV-2, negative serum (NS) from unvaccinated BFF, or monoclonal antibody (MAB) against SARS-CoV-2 and were then challenged with SARS-CoV-2. Lung grade, brain grade and total grade are listed relative to lung viral loads (CDC N2 Ct) as well as the ELISA titer of the serum that was transferred.

Mouse ID	BFF ID	Treatment	ELISA titer	CDC N2 Ct	CPE	Lung Grade	Brain Grade	Total Grade
1	8593 -3	PS	2560	20.993	Pos	1	0	1
2	8593 -12	PS	640	21.150	Pos	1	0	1
3	8847 -2	PS	10240	16.967	Pos	3	1	4
4	8847 -12	PS	2560	24.253	Pos	0	0	0
6	8995 -12	PS	10240	17.390	Pos	0	2	2
8	9135 -12	PS	2560	20.920	Pos	3	0	3
9	9189 -3	PS	10240	20.940	Pos	2	0	2
10	9189 -12	PS	2560	19.970	Pos	4	0	4
11	9219 -3	PS	10240	37.788	Neg	1	0	1
12	9219 -12	PS	10240	21.213	Pos	2	0	2
14	9337 -12	PS	10240	14.824	Pos	3	0	3
16	9371 -12	PS	2560	19.801	Pos	3	0	3
17	9422 -3	PS	40960		Neg	0	0	0
18	9422 -12	PS	2560	23.401	Pos	1	2	3
19	9469 -3	PS	10240	33.596	Neg	0	1	1
20	9469 -12	PS	2560	20.113	Pos	0	1	1
21	9476 -2	PS	10240	24.980	Pos	1	0	1
22	9476 -12	PS	2560	18.166	Pos	5	0	5
23	9568 -3	PS	10240	22.666	Pos	4	0	4

Immunogenicity, safety, and anti-viral efficacy of a subunit SARS-CoV-2 vaccine candidate in captive black-footed ferrets (*Mustela nigripes*) and their susceptibility to viral challenge.

Ariel E. Leon [1], Della Garelle [2], Airn Hartwig [3], Elizabeth A. Falendysz [1], Hon S. Ip [1], Julia S. Lankton [1],
Tyler N. Tretten [2], Terry R. Spraker [4], Richard Bowen [3,†], Tonie E. Rocke [1,*,†]

24	9568 -12	PS	2560	20.829	Pos	3	0	3
25	9571 -2	PS	40960	29.669	Neg	1	0	1
26	9571 -12	PS	2560	15.902	Pos	5	0	5
28	9576 -12	PS	160	15.113	Pos	5	0	5
29	9598 -3	PS	2560	19.735	Pos	1	3	4
30	9598 -12	PS	0	15.937	Pos	1	0	1
31	8710 -3	NS	160	22.153	Pos	5	0	5
32	8710 -12	NS	160	18.972	Pos	1	1	2
34	8721 -12	NS	160	16.448	Pos	0	1	1
35	8970 -2	NS	160	18.673	Pos	3	2	5
36	8970 -12	NS	160	22.603	Pos	1	0	1
38	9247 -12	NS	160	18.836	Pos	1	0	1
40	9311 -12	NS	160	20.337	Pos	1	0	1
46	9519 -12	NS	160	20.146	Pos	4	0	4
47	9592 -3	NS	160	19.587	Pos	3	0	3
48	9592 -12	NS	160	20.690	Pos	3	0	3
49	20 ug Ab	MAB		26.439	Neg	1	0	1
50	20 ug Ab	MAB		29.120	Neg	1	0	1
51	20 ug Ab	MAB		26.645	Neg	1	0	1
52	20 ug Ab	MAB		39.650	Neg	1	0	1
53		PBS		Not done	Neg	0	0	0
54		PBS		Not done	Neg	0	0	0

Immunogenicity, safety, and anti-viral efficacy of a subunit SARS-CoV-2 vaccine candidate in captive black-footed ferrets (*Mustela nigripes*) and their susceptibility to viral challenge.

Ariel E. Leon [1], Della Garelle [2], Airn Hartwig [3], Elizabeth A. Falendysz [1], Hon S. Ip [1], Julia S. Lankton [1],
Tyler N. Tretten [2], Terry R. Spraker [4], Richard Bowen [3,†], Tonie E. Rocke [1,*,†]

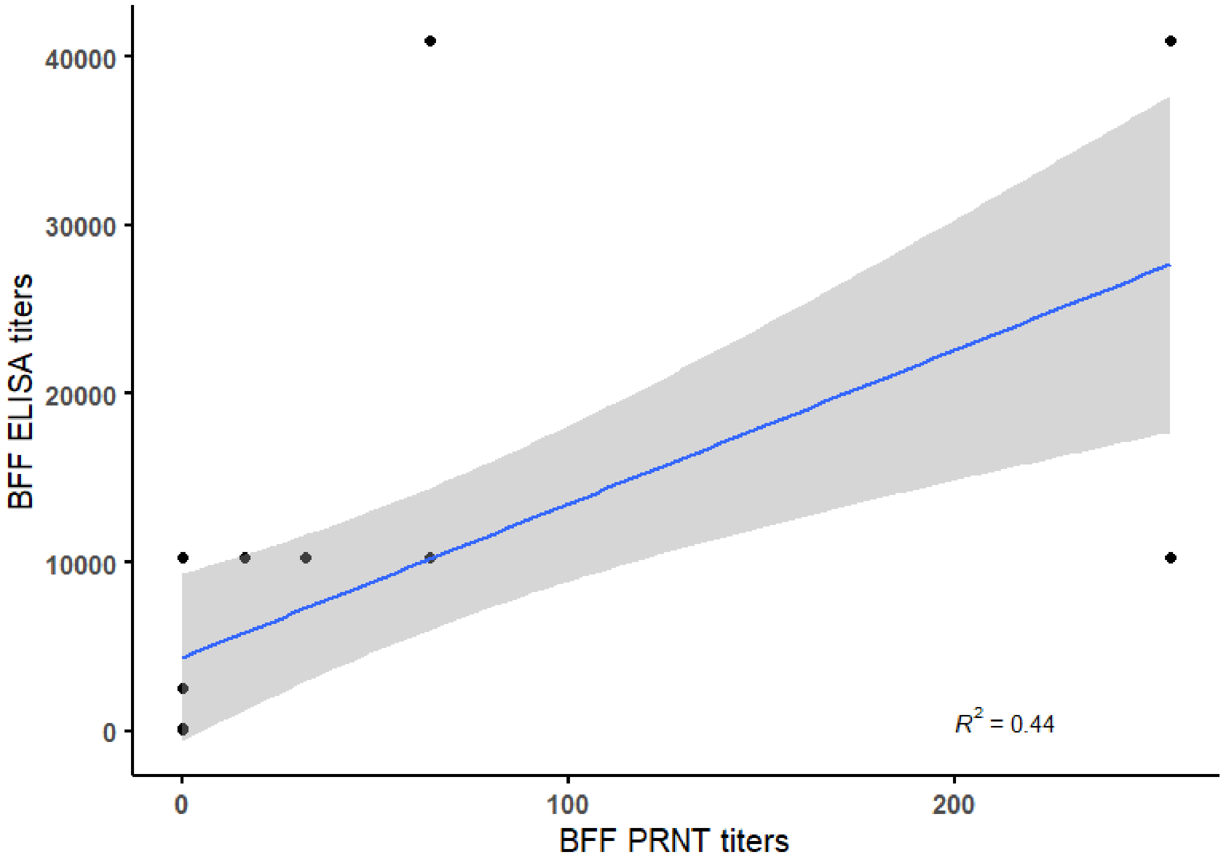

Figure S1. SARS-CoV-2 vaccinated black footed-ferret (*Mustela nigripes*; BFF) anti-S1 SARS-CoV-2 enzyme linked immunosorbent assay (ELISA) titers by their plaque reduction neutralization ($PRNT_{50}$) titers at 2-3 weeks post-boost. Each point represents an individual. The line represents a best fit linear regression, and shading represents the standard error of that regression.

Immunogenicity, safety, and anti-viral efficacy of a subunit SARS-CoV-2 vaccine candidate in captive black-footed ferrets (*Mustela nigripes*) and their susceptibility to viral challenge.

Ariel E. Leon [1], Della Garelle [2], Airn Hartwig [3], Elizabeth A. Falendysz [1], Hon S. Ip [1], Julia S. Lankton [1], Tyler N. Tretten [2], Terry R. Spraker [4], Richard Bowen [3,†], Tonie E. Rocke [1,*,†]

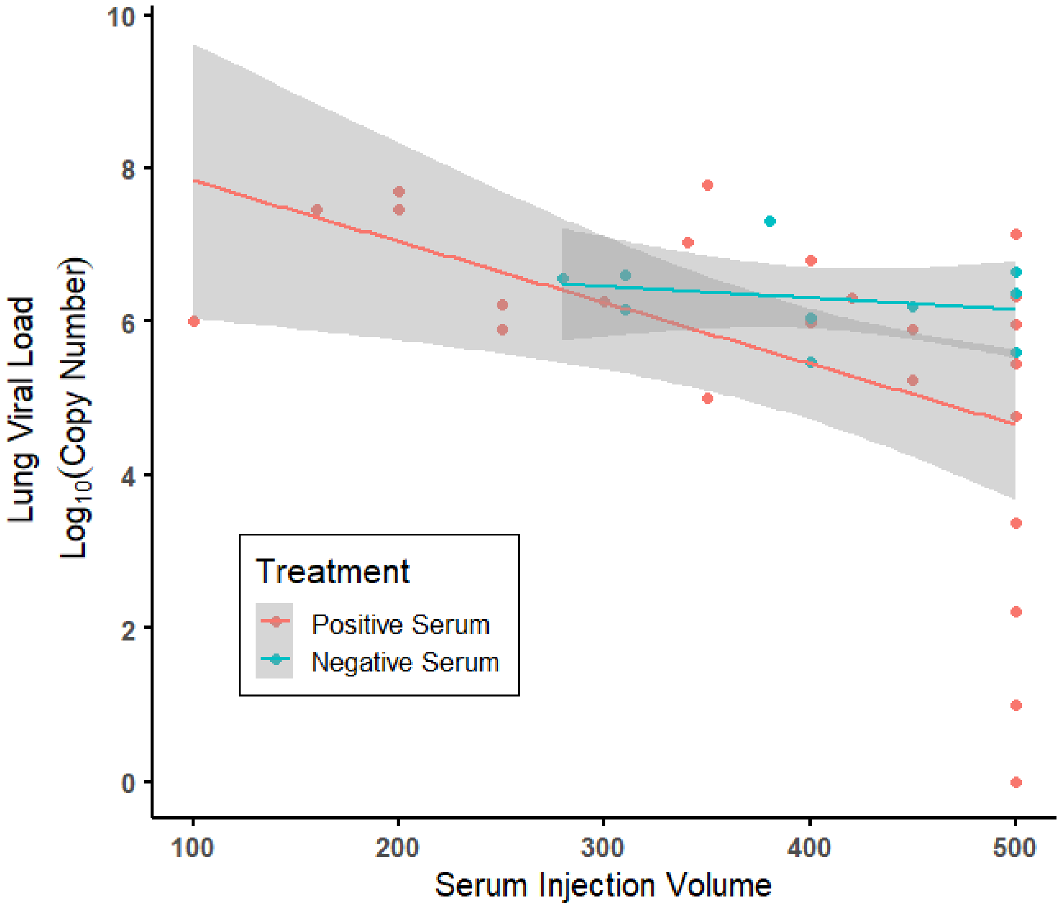

Figure S2. Lung viral load serum by intraperitoneal injection serum volume for mice treated with either black-footed ferret (*Mustela nigripes*; BFF) serum positive for anti-S1ARS-CoV-2 antibodies, or BFF serum negative for anti-S1 SARS-CoV-2 antibodies. The lines represent best fit linear regressions, and shading represents the standard error of that regression.

Immunogenicity, safety, and anti-viral efficacy of a subunit SARS-CoV-2 vaccine candidate in captive black-footed ferrets (*Mustela nigripes*) and their susceptibility to viral challenge.

Ariel E. Leon [1], Della Garelle [2], Airn Hartwig [3], Elizabeth A. Falendysz [1], Hon S. Ip [1], Julia S. Lankton [1], Tyler N. Tretten [2], Terry R. Spraker [4], Richard Bowen [3,†], Tonie E. Rocke [1,*,†]

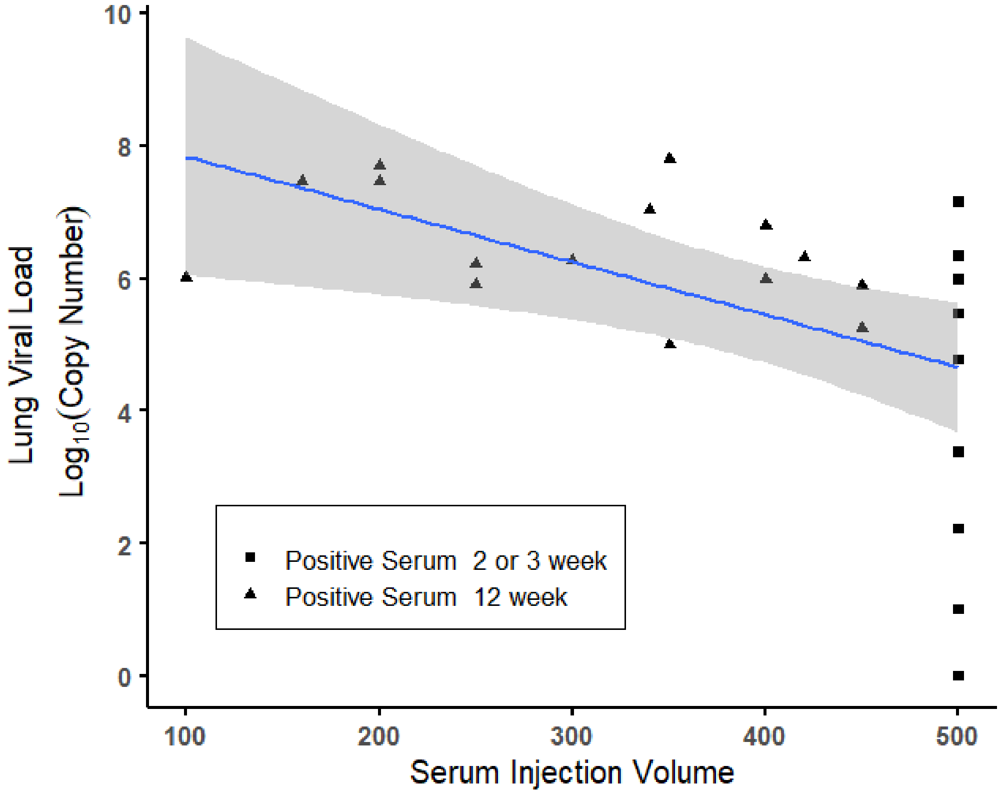

Figure S3. Lung viral load serum by intraperitoneal injection serum volume for mice treated with black-footed ferret (*Mustela nigripes*) serum positive for anti-S1 SARS-CoV-2 antibodies, either 2-3 week (represented by square points) or 12-week post-boost (triangular points). The line represents a best fit linear regression, shading representing the standard error of that regression.

Immunogenicity, safety, and anti-viral efficacy of a subunit SARS-CoV-2 vaccine candidate in captive black-footed ferrets (*Mustela nigripes*) and their susceptibility to viral challenge.

Ariel E. Leon [1], Della Garelle [2], Airn Hartwig [3], Elizabeth A. Falendysz [1], Hon S. Ip [1], Julia S. Lankton [1], Tyler N. Tretten [2], Terry R. Spraker [4], Richard Bowen [3,†], Tonie E. Rocke [1,*,†]

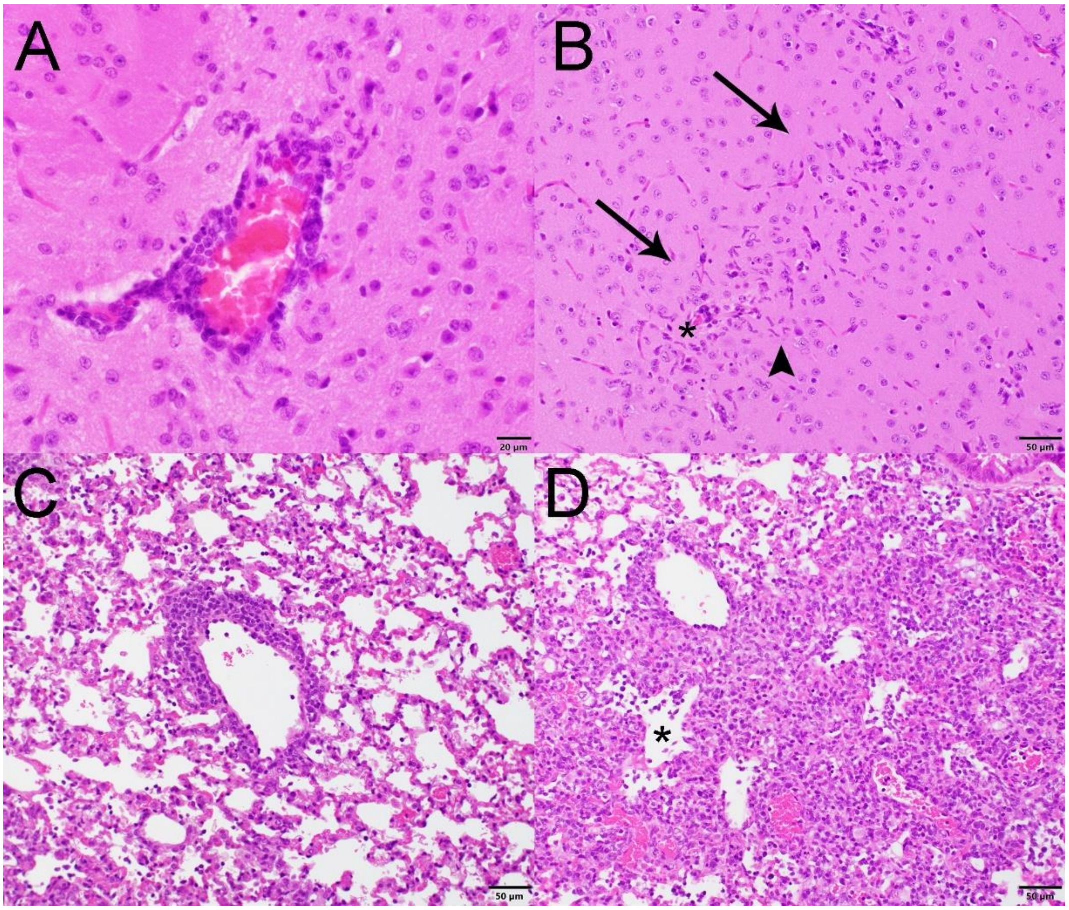

Figure S4. Photomicrographs of lesions in K18-hACE-2 mice inoculated intraperitoneally with serum from SARS-CoV-2 vaccinated (A and B) and control (C and D) black-footed ferrets (*Mustela nigripes;* BFF) and inoculated intranasally with SARS-CoV-2. (A) Brain: A blood vessel is surrounded by low numbers of lymphocytes, plasma cells, and histiocytes (perivascular cuffing). Hematoxylin and eosin stain. (B) Brain: An area of gliosis (arrows) is characterized by hypercellularity with many elongate microglial nuclei (arrowhead). A small blood vessel with few adjacent mononuclear cells is within this area (asterisk). Hematoxylin and eosin stain. (C) Lung: An interstitial blood vessel is surrounded by low numbers of lymphocytes, plasma cells, and histiocytes. Hematoxylin and eosin stain. (D) Lung: The majority of alveolar septa are expanded by large numbers of lymphocytes, plasma cells, and histiocytes. Similar inflammatory cells are within remaining alveolar spaces (asterisk). Hematoxylin and eosin stain.

Immunogenicity, safety, and anti-viral efficacy of a subunit SARS-CoV-2 vaccine candidate in captive black-footed ferrets (*Mustela nigripes*) and their susceptibility to viral challenge.

Ariel E. Leon [1], Della Garelle [2], Airn Hartwig [3], Elizabeth A. Falendysz [1], Hon S. Ip [1], Julia S. Lankton [1], Tyler N. Tretten [2], Terry R. Spraker [4], Richard Bowen [3,†], Tonie E. Rocke [1,*,†]

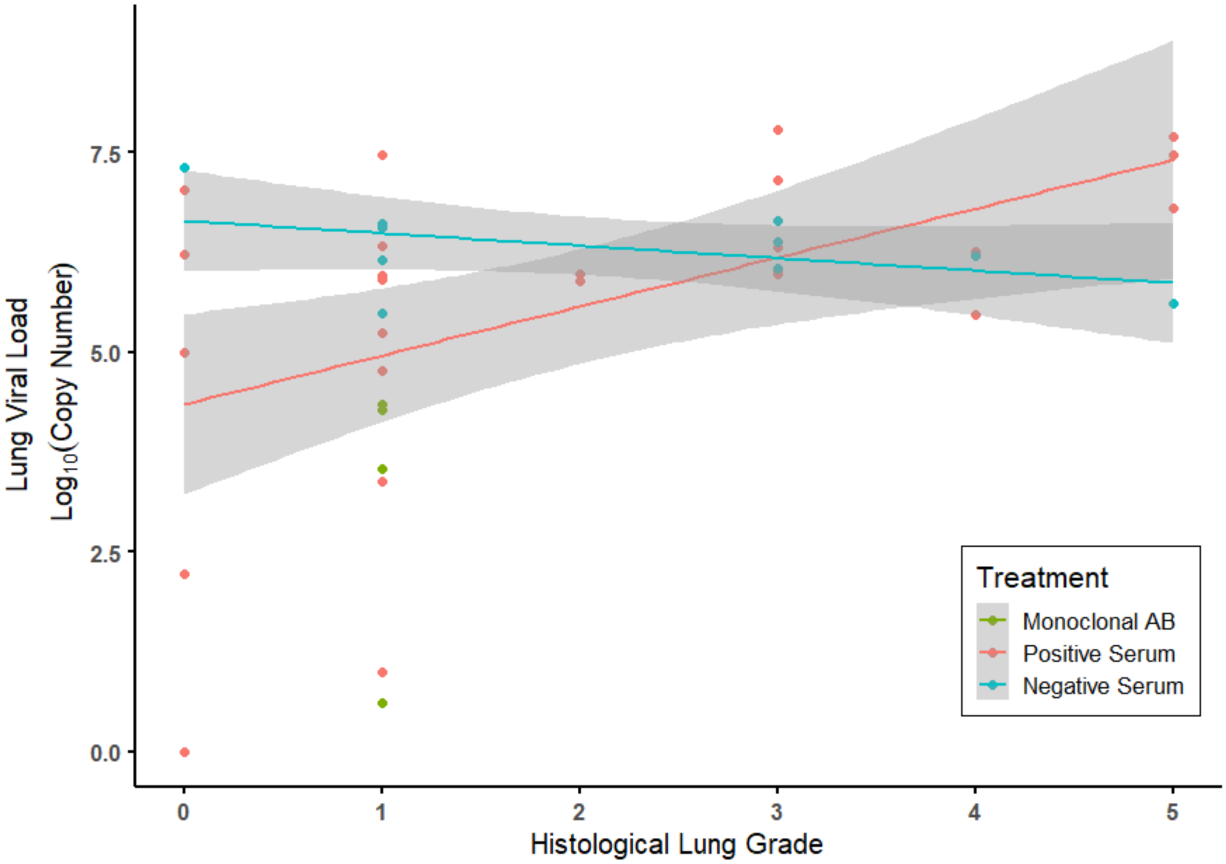

Figure S5. Lung viral load by histological lung grade for mice treated with either Monoclonal antibodies (AB), black-footed ferret (*Mustela nigripes*; BFF) serum positive for anti-S1 SARS-CoV-2 antibodies, or BFF serum negative for anti-S1 SARS-CoV-2 antibodies. The lines represent best fit linear regressions, and shading represents the standard error of that regression.

Immunogenicity, safety, and anti-viral efficacy of a subunit SARS-CoV-2 vaccine candidate in captive black-footed ferrets (*Mustela nigripes*) and their susceptibility to viral challenge.

Ariel E. Leon [1], Della Garelle [2], Airn Hartwig [3], Elizabeth A. Falendysz [1], Hon S. Ip [1], Julia S. Lankton [1], Tyler N. Tretten [2], Terry R. Spraker [4], Richard Bowen [3,†], Tonie E. Rocke [1,*,†]

A B

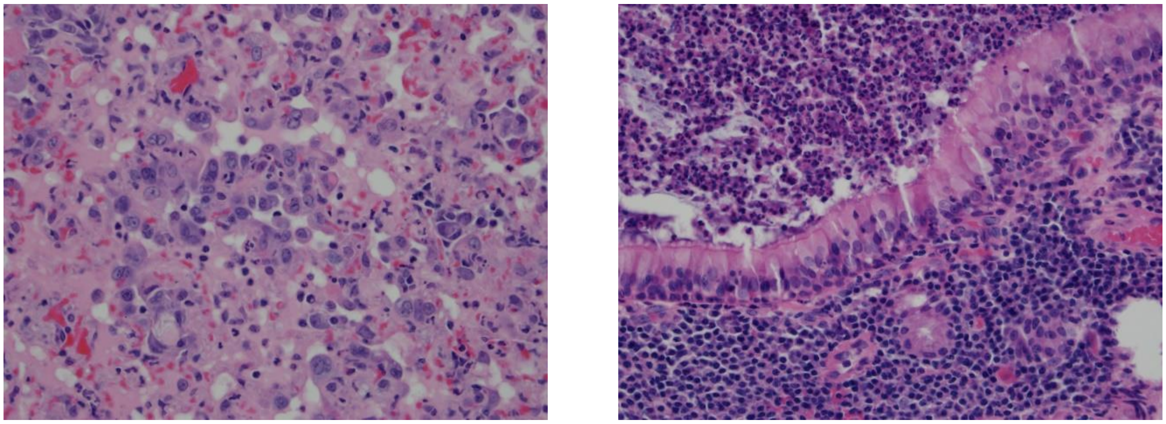

Figure S6. Photomicrographs of lung and nasal cavity of black-footed ferrets (*Mustela nigripes*; BFF) examined at days 3 and 14 post-inoculation (PI) with SARS-CoV-2. Histological lesion in the lungs were patchy areas of interstitial pneumonia and were found in all three BFF euthanized at 3 days PI and in one ferret euthanized at 14 days PI (A). Suppurative rhinitis was found in three of the BFF, one euthanized at day 3 PI and two at 14 days PI (B).
